# Normal bacterial flora may inhibit *Candida albicans* biofilm formation by Autoinducer-2

**DOI:** 10.3389/fcimb.2014.00117

**Published:** 2014-08-28

**Authors:** Robert J. C. McLean

**Affiliations:** Department of Biology, Texas State UniversitySan Marcos, TX, USA

**Keywords:** Autoinducer-2, Interkingdom signaling, biofilm inhibition, Quorum Sensing, Candidiasis

Bachtiar et al. (2014) present a very interesting study in which they illustrate the ability of AI-2, produced by *Aggregatibacter actinomycetemcomitans* to inhibit biofilm formation by the fungal opportunistic pathogen *Candida albicans*. *C. albicans* is considered to be the most common fungal pathogen of humans, manifesting itself in the oral cavity (oral candidiasis or thrush), female reproductive tract (commonly referred to as a yeast infection), and less commonly in the digestive tract. It is also a notable blood-borne nosocomial pathogen (reviewed in Moyes and Naglik, 2011). While candidiasis is a complication in immunocompromised patients (Moyes and Naglik, 2011; Gow and Hube, 2012), in most other situations candidiasis arises as a consequence of normal flora disruption (Gow and Hube, 2012).

In contrast to the considerable work done on mucosal immunity and other aspects of the immune system, less work has been done on mechanisms whereby normal flora control *C. albicans*. As part of its virulence, *C. albicans* does form biofilms through a quorum-regulated developmental process analogous to bacterial biofilms (Chandra et al., 2001; Ramage et al., 2002). Hogan and Kolter (2002) showed that *Pseudomonas aeruginosa* biofilms could colonize and inhibit *C. albicans* only when *C. elegans* was growing in the mycelia form and not the yeast form. These authors found that *P. aeruginosa* production of type 4 pili, involved in biofilm formation, antimicrobial phenazines, and other virulence factors, notably phospholipase C, were important inhibitors of *C. albicans* (Hogan and Kolter, 2002). Cruz et al. illustrated how proteins produced by *Enterococcus faecalis* could inhibit *C. albicans* hyphal growth and virulence (Cruz et al., 2013). There have also been reports of other specific interactions between individual bacteria and *Candida albicans* (reviewed in Shirtliff et al., 2009). While these studies address specific mechanisms, relevant to a specific bacterial species' interaction with *C. albicans*, it does not address the general phenomenon of normal flora inhibition of *C. albicans* growth and virulence. In this context, the study by Bachtiar et al. (2014), represents an intriguing and very welcome development.

Autoinducer 2 (AI-2), encoded by *luxS* has been likened to a universal quorum signal in that it is produced and responded to by a wide number of bacterial species (Ng and Bassler, 2009). In their study, Bachtiar et al. (2014) focused on the interaction of *A. actinomycetemcomitans* with *C. albicans*. Using genetically defined strains, they showed that *luxS* mutants were unable to inhibit biofilm formation in *C. albicans*, but that this inhibition could be restored by genetic complementation or through the addition of physiologically-relevant concentrations of synthetic AI-2 or spent media from AI-2-producing *A. actinomycetemcomitans*. Although *A. actinomycetemcomitans*, found in the sub-gingival crevice, is normally a minor component of the oral cavity (Ramsey and Whiteley, 2009), the universal presence of AI-2 in many bacteria (Ng and Bassler, 2009) suggests that this signal molecule may represent a heretofore unrecognized general mechanism of the normal flora that protect against *C. albicans* infections. Certainly, work is needed with other AI-2-producing members of the normal flora to confirm this possibility. In light of the work by Hogan and Kolter (2002), it would also be very appropriate to determine whether AI-2 inhibition of *C. albicans* was a general phenomenon rather than being restricted to a specific physiological state (i.e., mycelia or yeast growth) of this fungal opportunistic pathogen.

In summary, while there is considerable interest in exploiting quorum signal disruption as an antimicrobial therapy (Christensen et al., 2012; Vega et al., 2014), the work of Bachtiar et al. (2014) implies that enhanced *C. albicans* virulence might be an undesired consequence of this quorum-inhibiting strategy, as is the case with antibiotic-induced normal flora reduction (Gow and Hube, 2012).

## Conflict of interest statement

The author declares that the research was conducted in the absence of any commercial or financial relationships that could be construed as a potential conflict of interest.

## References

[B1] Bachtiar E. W. Bachtiar B. M. Jarosz L. M. Amir L. R. Sunarto H. Ganin H. (2014). AI-2 of *Aggregatibacter actinomycetemcomitans* inhibits *Candida albicans* biofilm formation. Front. Cell. Infect. Microbiol. 4:94 10.3389/fcimb.2014.00094 25101248 PMC4104835

[B2] Chandra J. Kuhn D. M. Mukherjee P. K. Hoyer L. L. McCormick T. Ghannoum M. A. (2001). Biofilm formation by the fungal pathogen *Candida albicans*: development, architecture, and drug resistance. J. Bacteriol. 183, 5385–5394 10.1128/JB.183.18.5385-5394.2001 11514524 PMC95423

[B3] Christensen L. D. van Gennip M. Jakobsen T. H. Alhede M. Hougen H. P. Hoiby N. (2012). Synergistic antibacterial efficacy of early combination treatment with tobramycin and quorum-sensing inhibitors against *Pseudomonas aeruginosa* in an intraperitoneal foreign-body infection mouse model. J. Antimicrob. Chemother. 67, 1198–1206 10.1093/jac/dks002 22302561

[B4] Cruz M. R. Graham C. E. Gagliano B. C. Lorenz M. C. Garsin D. A. (2013). *Enterococcus faecalis* inhibits hyphal morphogenesis and virulence of *Candida albicans*. Infect. Immun. 81, 189–200 10.1128/IAI.00914-12 23115035 PMC3536143

[B5] Gow N. A. Hube B. (2012). Importance of the *Candida albicans* cell wall during commensalism and infection. Curr. Opin. Microbiol. 15, 406–412 10.1016/j.mib.2012.04.005 22609181

[B6] Hogan D. A. Kolter R. (2002). *Pseudomonas-Candida* interactions: an ecological role for virulence factors. Science 296, 2229–2232 10.1126/science.1070784 12077418

[B7] Moyes D. L. Naglik J. R. (2011). Mucosal immunity and *Candida albicans* infection. Clin. Dev. Immunol. 2011:346307 10.1155/2011/346307 21776285 PMC3137974

[B8] Ng W. L. Bassler B. L. (2009). Bacterial quorum-sensing network architectures. Annu. Rev. Genet. 43, 197–222 10.1146/annurev-genet-102108-134304 19686078 PMC4313539

[B9] Ramage G. Saville S. P. Wickes B. L. López-Ribot J. L. (2002). Inhibition of *Candida albicans* biofilm formation by farnesol, a quorum-sensing molecule. Appl. Environ. Microbiol. 68, 5459–5463 10.1128/AEM.68.11.5459-5463.2002 12406738 PMC129887

[B10] Ramsey M. M. Whiteley M. (2009). Polymicrobial interactions stimulate resistance to host innate immunity through metabolite perception. Proc. Natl. Acad. Sci. U.S.A. 106, 1578–1583 10.1073/pnas.0809533106 19164580 PMC2629492

[B11] Shirtliff M. E. Peters B. M. Jabra-Rizk M. A. (2009). Cross-kingdom interactions: *Candida albicans* and bacteria. FEMS Microbiol. Rev. 299, 1–8 10.1111/j.1574-6968.2009.01668.x 19552706 PMC4406406

[B12] Vega L. M. Alvarez P. J. McLean R. J. C. (2014). Bacterial signaling ecology and potential applications during aquatic biofilm construction. Microb. Ecol. 68, 24–34 10.1007/s00248-013-0321-1 24276538

